# Role and prognostic value of N6-methyladenosine RNA modification regulators in glycolytic reprogramming during sepsis

**DOI:** 10.1186/s40560-026-00920-4

**Published:** 2026-07-30

**Authors:** Xia Liu, Yanyu Zhang, Lei Feng, Aruna Xiang, Zhuqing Kang, Qi Zhang, Lei Kang

**Affiliations:** 1 Department of Critical Care Medicine, Peking University Cancer Hospital (Inner Mongolia Campus), Hohhot, Inner Mongolia China; 2 Department of Cardiology, Peking University Cancer Hospital (Inner Mongolia Campus), Hohhot, Inner Mongolia China

**Keywords:** N6-methyladenosine, Sepsis, Glycolysis, ALKBH5, Prognostic model, Epitranscriptome

## Abstract

**Objective:**

This study aims to investigate the expression profiling characteristics of N6-methyladenosine (m6A) RNA modification regulators in sepsis, their associations with glycolytic metabolic reprogramming, and their preliminary prognostic value.

**Methods:**

We integrated multiple sepsis transcriptomic cohorts (GSE65682, GSE95233, GSE54514) from the GEO database to systematically analyze the differential expression of 15 m6A regulators. Gene Set Variation Analysis (GSVA) scores, weighted gene co-expression network analysis (WGCNA) networks, and Spearman correlation analyses were utilized to assess the associations between m6A regulators and glycolytic activity. The regulation of glycolytic genes by m6A modifications was confirmed using ALKBH5 knockout models (GSE198316, GSE224650) and MeRIP-seq data (GSE225143). A prognostic model was developed by integrating CIBERSORT immune cell infiltration analysis, single-cell RNA sequencing data (GSE147363), and LASSO-Cox regression.

**Results:**

Most m6A regulators are significantly downregulated in patients with sepsis, while IGF2BP2 and IGF2BP3 are upregulated. ALKBH5 clusters with IGF2BP2 within the same WGCNA module and exhibits a positive correlation with glycolytic activity (rho=0.592, *P*=1.0 × 10⁻^29^). The differentially expressed genes (DEGs) resulting from ALKBH5 knockout significantly overlap with sepsis-associated DEGs (*n*=124; OR=2.48, *P*=5.10 × 10⁻^11^), which are enriched in the HIF-1 signaling pathway and glycolytic processes. Independent validation confirmed a directional consistency of 89.6%. MeRIP-seq data indicated m6A-mediated associations with glycolytic genes, including ENO1, GAPDH, HK2, and LDHA. Single-cell analysis demonstrated a positive correlation between ALKBH5 and most glycolytic genes in CD14+ monocytes. A prognostic model based on four m6A genes (HNRNPC, YTHDF1, YTHDF2, VIRMA) achieved a C-index of 0.750 in the training set and 0.709–0.718 in external validation sets.

**Conclusion:**

These integrated analyses suggest that ALKBH5-associated m6A modification is correlated with sepsis-associated glycolytic reprogramming and support a biologically plausible, hypothesis-generating ALKBH5-associated glycolytic program that requires prospective and experimental validation. The m6A-based four-gene model showed preliminary prognostic potential for 28-day mortality, but its external reproducibility was incomplete and requires validation in larger, prospectively collected and clinically harmonized cohorts.

**Supplementary Information:**

The online version contains supplementary material available at https://doi.org/10.1186/s40560-026-00920-4.

## Introduction

Sepsis is a life-threatening organ dysfunction caused by dysregulated host response to infection, ranking among the leading causes of death in intensive care unit (ICU) patients [1–3]. Globally, approximately 49 million sepsis cases occur annually, resulting in about 11 million deaths, accounting for nearly 20% of all global deaths [4]. Despite recent advances in anti-infective therapies and organ support, sepsis mortality remains high, with 28-day mortality rates reaching 25–30% [5]. The pathophysiology of sepsis is highly complex, encompassing immune-inflammatory dysregulation, coagulopathy, metabolic reprogramming, and multi-organ failure [6, 7 ]. Among these, metabolic reprogramming has been identified as a core driver of immune cell dysfunction in sepsis. During the early stages, innate immune cells undergo a metabolic shift from oxidative phosphorylation to aerobic glycolysis—the so-called Warburg effect—which provides the energy foundation for rapid cytokine production and bactericidal functions [8, 9 ]. However, sustained excessive glycolytic activation leads to immune exhaustion and organ damage. The imbalance between glycolysis and oxidative phosphorylation is considered a critical transition point from the early hyperinflammatory state to the late immunosuppressive phase of sepsis [10, 11 ]. Therefore, elucidating the regulatory mechanisms of glycolytic metabolic reprogramming in sepsis is crucial for understanding the disease's essence and identifying therapeutic targets.

N6-methyladenosine (m6A) is the most prevalent internal mRNA modification and is dynamically deposited by writers (e.g., METTL3, METTL14, and WTAP), removed by erasers (FTO and ALKBH5), and decoded by readers (YTH-domain and IGF2BP proteins) to control mRNA splicing, export, translation, and stability [12–18]. In immunity and metabolism, m6A regulates dendritic-cell and T-cell function and macrophage inflammatory responses [19–22], and, importantly, directly tunes the stability and translation of key glycolytic enzymes (e.g., HK2, PKM2, LDHA) in tumors [23, 24 ]. However, the precise mechanism by which m6A drives glycolytic reprogramming during sepsis remains unclear.

Particularly in the context of metabolic reprogramming during sepsis, whether m6A-associated regulatory programs are linked to immunometabolic phenotypes through epitranscriptomic changes in glycolysis-related genes remains incompletely understood [25, 26 ]. In addition, the predictive value of m6A-based prognostic models in sepsis requires cautious evaluation across independent and clinically heterogeneous cohorts. With the ongoing advancement of high-throughput sequencing technologies and the expansion of public data resources, integrating transcriptomic data from multiple independent cohorts with ALKBH5 perturbation data and MeRIP-seq epitranscriptomic data provide a feasible strategy to generate mechanistic hypotheses regarding m6A-associated metabolic dysregulation in sepsis [27].

In addition, we evaluate whether independent ALKBH5 perturbation datasets (GSE198316 and GSE224650) and m6A epitranscriptomic datasets (GSE225143, GSE201060, and GSE127732) support a potential ALKBH5-associated glycolytic program at transcriptomic and epitranscriptomic levels. By integrating CIBERSORT-based immune cell infiltration analysis with single-cell RNA sequencing data (GSE147363), we explore the associations among m6A regulators, glycolysis, and the immune microenvironment. Finally, an m6A regulator-based prognostic model was constructed using LASSO-Cox regression, and its predictive performance and external reproducibility were evaluated across validation cohorts. This study is expected to offer a hypothesis-generating epitranscriptomic perspective on metabolic-immune dysregulation in sepsis and to provide a preliminary basis for future validation studies.

## Materials and methods

### Data sources and preprocessing

All datasets were log-transformed and quantile-normalized. Platform-related batch effects were mitigated using ComBat with disease condition included as a protected covariate to preserve sepsis-control signal. The originally reported PC1-associated variance decreased from 70.96% before correction to 15.71% after correction. To avoid relying on this metric alone, we further evaluated cross-platform harmonization using PCA visualization, kBET, silhouette width, PERMANOVA, per-gene variance decomposition, and within-cohort logFC preservation analyses. Quality control indicated no missing values in any dataset, supporting the reliability of the data (Figure S1).

We further examined the temporal sampling characteristics of each cohort, because sepsis is a dynamic syndrome whose transcriptomic profile evolves from the hyperinflammatory to the immunosuppressive phase. GSE65682 and GSE95233 sampled whole blood within 24–48 h of intensive-care-unit admission, whereas GSE54514 provided longitudinal sampling between days 1 and 5; to minimize the influence of disease stage we restricted all cross-cohort comparisons to the earliest available time point per patient and treated sampling day as a covariate in sensitivity analyses. Residual platform heterogeneity after ComBat correction was quantified by the proportion of variance explained by the first principal component (70.96% before versus 15.71% after correction), and genes with incomplete probe coverage across platforms (METTL14 and VIRMA) were analyzed only within cohorts in which they were reliably detected, as detailed in Sect. “Definition of m6A regulators and differential expression analysis”.

Because this study integrated nine independent public datasets that differ in their original ethical frameworks, sepsis definitions (Sepsis-2 versus Sepsis-3), recruiting settings (single- versus multi-center), and age ranges (adult versus mixed cohorts), we explicitly verified the ethical provenance of each source. Every original study had been approved by its respective institutional review board and had obtained informed consent in accordance with the Declaration of Helsinki (Supplementary Table S1). As the present work constitutes a secondary analysis of fully de-identified, openly accessible data, it was exempt from additional institutional review board approval under the regulations governing non-interventional research on anonymized data, and this exemption was confirmed by the institutional ethics committee of the corresponding author's institution. No attempt was made to re-identify any participant, and datasets whose original consent terms were incompatible with secondary use were excluded.

### Definition of m6A regulators and differential expression analysis

This study initially included 20 known m6A regulatory factors (Writers: METTL3, METTL14, METTL16, WTAP, RBM15, RBM15B, VIRMA, ZC3H13; Erasers: FTO, ALKBH5; Readers: YTHDF1-3, YTHDC1-2, IGF2BP1-3, HNRNPC, HNRNPA2B1). After intersection matching with the annotation files of three GEO microarray platforms (GPL13667, GPL10558, GPL570), 14 genes were detectable across all three datasets and were included in differential expression analysis and cross-cohort validation. METTL14 did not enter the intersection of the three platforms due to incomplete probe coverage; however, METTL14 could be detected in the primary GSE65682 cohort through probe mapping and was included in the single-cohort association analysis.

### Survival analysis and prognostic association

In the GSE65682 cohort, patients were stratified into high- and low-expression groups using the median expression level as the cutoff. Survival curves were plotted using the Kaplan–Meier method, and the log-rank test was employed to compare 28-day survival differences between groups. Univariate Cox proportional hazards models were used to estimate hazard ratios (HRs) and 95% confidence intervals (CIs) for each m6A regulator. Spearman's rank correlation analysis was performed to evaluate the association between m6A regulator expression levels and 28-day mortality.

Sepsis is a markedly heterogeneous syndrome, and the integrated cohorts encompassed differences in infection source, causative organism, disease severity, shock status, organ dysfunction (acute kidney injury, acute respiratory distress syndrome, and disseminated intravascular coagulation), age, and treatment. Where the original metadata permitted, we performed stratified sensitivity analyses by the available severity indicators and adjusted the prognostic comparisons for age; however, harmonized clinical covariates such as the SOFA score, infection source, and treatment exposure were not uniformly reported across cohorts, which constrains full confounder adjustment and is acknowledged as a limitation.

To address this issue more transparently, we systematically summarized all available clinical metadata for each included cohort, including platform, sample source, sepsis/control composition, sampling time, age, sex, outcome, infection-related variables, disease severity proxies, organ dysfunction variables, and treatment-related information when available (Supplementary Table S2). Variables available within a given cohort were incorporated into sensitivity or stratified analyses where appropriate. Variables not reported in the public metadata were explicitly listed as unavailable and treated as potential sources of residual unmeasured confounding rather than assumed to be controlled.

### Glycolytic activity assessment and m6A–glycolysis association analysis

Glycolytic pathway activity scores for each sample were calculated using GSVA [28]. Spearman’s rank correlation was used to assess pairwise correlations between m6A regulators and glycolytic genes (FDR<0.001). A co-expression network between m6A regulators and glycolysis-related genes was constructed using WGCNA [29]; the optimal soft-thresholding power was selected, co-expression modules were identified, and the m6A regulators most closely associated with glycolytic function were determined by correlating module eigengenes with glycolytic activity scores.

### Functional validation of ALKBH5 and multi-omics integration analysis

Two independent ALKBH5-knockout transcriptomic datasets were retrieved from the GEO database: GSE198316 (batch 1; ALKBH5 knockout vs. control macrophages) and GSE224650 (batch 2; independent laboratory replication). A cross-analysis was performed between differentially expressed genes in the ALKBH5 knockout model (|log2FC|> 1, adjusted P<0.05) and sepsis-associated genes, using Fisher's exact test and the hypergeometric test to assess the significance of overlap. Enrichment analyses for overlapping genes were conducted for GO Biological Process terms and KEGG pathways using Enrichr [30]. A direction-consistency analysis was then performed to evaluate whether the directions of expression changes for genes shared between the ALKBH5 knockout and sepsis were concordant. The criteria used for data extraction from each dataset (platform, group definition, inclusion thresholds) were pre-specified, and dataset validity was evaluated before downstream integration.

### Validation of m6A modification

We integrated three epitranscriptomic datasets (GSE225143: MeRIP-seq in METTL3 conditional knockout vs. wild-type mouse macrophages; GSE201060: m6A-seq in human cell lines; GSE127732: m6A-seq in mouse tissues) to validate m6A-mediated regulatory effects at the epitranscriptome level. Identified m6A modification peaks at the DRACH motif (D=A/G/U, R=A/G, H=A/C/U) on glycolytic genes and assessed changes in m6A modification levels following METTL3 knockout. Simultaneously, the m6A2Target database was queried to obtain a list of validated ALKBH5 target genes, and three-way cross-validation was performed with the differentially expressed genes identified in this study [31].

### Analysis of the immune microenvironment

The CIBERSORT algorithm was applied to the GSE65682 cohort to perform immune cell composition deconvolution, estimating the relative proportions of 22 immune cell subpopulations [32]. Differences in immune cell composition between sepsis patients and healthy controls were assessed, and variations in immune cell infiltration were analyzed between ALKBH5 high- and low-expression groups. The correlation between neutrophil proportion and glycolytic activity score was evaluated, as was the association between the proportion of each immune cell subset and glycolytic activity. Enrichment scores for hypoxia pathways, oxidative phosphorylation, and inflammatory response pathways were calculated using GSVA to examine the associations between m6A regulators and multiple metabolic-immune pathways.

### Single-cell RNA sequencing validation

The single-cell RNA sequencing dataset GSE147363 for sepsis (comprising 38,559 cells from whole blood PBMC samples of sepsis patients and healthy controls) was obtained from the GEO database. Standardization, principal component analysis (PCA), and UMAP dimensionality reduction visualization were performed using the Scanpy toolkit. Cell type annotation was conducted via the Leiden clustering algorithm. Expression patterns of m6A regulators and glycolytic genes were analyzed across cell subpopulations, with a focus on evaluating the co-expression relationship between ALKBH5 and glycolytic genes in different immune cell subpopulations. The proportion of ALKBH5-positive cells was compared between sepsis and healthy states.

To avoid pseudo-replication in the single-cell analyses, individual cells were not treated as statistically independent units; instead, correlation and proportion estimates were first computed within each patient and then aggregated across patients, and group comparisons were performed at the patient level using the number of patients as the effective sample size. Reported single-cell P-values therefore reflect inter-patient variability rather than the inflated significance that can arise from the large number of cells.

For single-cell correlation analyses, we reported both statistical significance and effect magnitude. Spearman correlation coefficients were interpreted according to their absolute magnitude, and weak correlations were not considered sufficient evidence for direct regulatory effects. Where applicable, patient-level bootstrapping was used to estimate 95% confidence intervals for correlation coefficients, and concordance with independent bulk ALKBH5-knockout datasets was used as supportive evidence rather than proof of causality.

### Construction and validation of prognostic models

In the GSE65682 cohort, the data were randomly split into training and internal validation sets at a 7:3 ratio. Using 28-day mortality as the endpoint, we first performed univariate Cox regression on 15 m6A regulators (*P*<0.05) for candidate feature selection, and subsequently applied LASSO-Cox regression for further feature selection and model building. Tenfold cross-validation was employed to determine the optimal penalty parameter λ. A risk score was calculated based on the selected m6A regulators, and patients were stratified into high-risk and low-risk groups using the median risk score as the cutoff. Model performance was evaluated with the concordance index (C-index), time-dependent ROC curve (AUC), and calibration curves. The model's generalization ability was assessed in the internal validation set and in external validation sets GSE95233 and GSE54514.

To mitigate over-fitting, feature dimensionality was deliberately constrained: candidate genes were first filtered by univariate Cox regression, LASSO-Cox penalization with the more conservative lambda.1se rule was applied under tenfold cross-validation, and the final model retained only four genes, yielding an events-per-variable ratio above the conventional threshold of ten. Model performance was assessed exclusively in independent external cohorts (GSE95233 and GSE54514) that were never used for training or feature selection.

To improve transparency regarding external reproducibility, we summarized cohort-level performance for the four-gene model across the training set, internal validation set, GSE95233, and GSE54514, including log-rank P-values, C-index values, and time-dependent AUCs (Supplementary Table S4). GSE54514 was interpreted separately because it differed in platform, sampling design and sample size.

### Statistical methods

All statistical analyses were performed using Python 3.12 and R 4.3 software. Comparisons of continuous variables between groups were conducted using Welch’s t-test or the Mann–Whitney U test, depending on the data distribution. Correlation analysis was performed using Spearman's rank correlation test. Multiple comparisons were corrected using the Benjamini–Hochberg method. Survival analysis was performed using the Kaplan–Meier method with the log-rank test. Enrichment analysis was performed using Fisher’s exact test. A two-sided *P*<0.05 was considered statistically significant. Correlation coefficients are reported to three decimal places throughout.

For analyses involving simultaneous comparisons across multiple genes or groups (e.g., differential expression analysis, GSVA comparisons between groups, and CIBERSORT-based intergroup comparisons of immune cells), the Benjamini–Hochberg method was applied to control the false discovery rate (FDR), and adjusted P-values (adj-P) were reported. For single hypothesis tests (e.g., univariate Cox regression, log-rank test), raw P-values were reported.

## Results

### Expression profile characteristics of m6A regulatory factors in sepsis

**Fig. 1:**
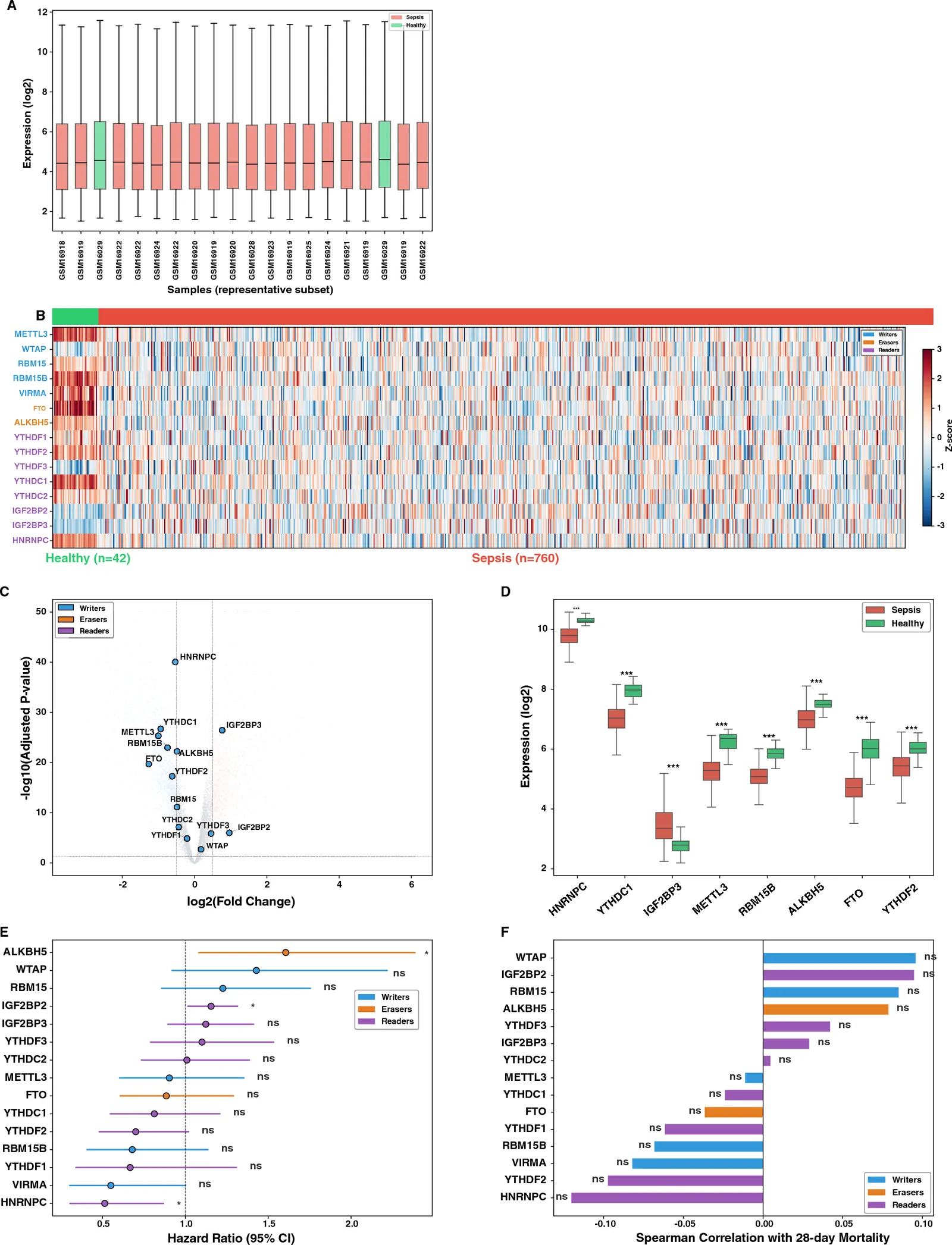
Expression profile of m6A regulators in sepsis. **A** Quality-control box plots of normalized expression across the three cohorts before and after ComBat correction; alignment of the post-correction distributions indicates that platform-related batch effects were substantially reduced. **B** Heatmap of the 14 cross-platform m6A regulators in GSE65682 (sepsis *n*=760, control *n*=42); columns are samples and rows are genes, with red denoting high and blue low scaled expression, illustrating coordinated down-regulation of most regulators in sepsis. **C** Volcano plot of differential expression (*x*-axis log2 fold-change, *y*-axis -log10 adjusted P); points to the right are upregulated (IGF2BP2/IGF2BP3) and to the left downregulated. **D** Box plots of the eight most significantly altered regulators between sepsis and controls. **E** Forest plot of univariate Cox hazard ratios for 28-day mortality; markers right of unity indicate risk and left of unity indicate protection. **F** Spearman correlation of each regulator with 28-day mortality. Together the panels show a globally remodeled m6A landscape with HNRNPC and ALKBH5 most strongly linked to outcome

Quality control analysis indicated good data quality across the three datasets, with platform-specific variations significantly reduced following ComBat batch correction (Fig. 1A and Supplementary Figure S1). In the main cohort GSE65682, heatmap analysis revealed that 14 m6A regulatory factors exhibited significant expression differences between sepsis patients (*n*=760) and healthy controls (*n*=42) (Fig. 1B). Volcano plot analysis revealed that most m6A regulators were significantly downregulated in sepsis, while IGF2BP3 (log2FC=0.76, adj-P=1.24×10⁻^27^) and IGF2BP2 (log2FC=0.962, adj-P=7.76×10⁻^7^) were significantly upregulated (Fig. 1C). Among these, HNRNPC was the most significantly downregulated (log2FC=− 0.53, adj-P=1.95×10⁻^41^), followed by METTL3 (log2FC=− 1.00, adj-P=1.35×10⁻^26^) and YTHDC1 (log2FC=− 0.94, adj-P=9.59×10⁻^28^). Box plots further illustrate the expression distributions of the eight m6A regulators with the most significant differences between sepsis and healthy controls (Fig. 1D). These differences were consistently validated in the GSE95233 validation cohort, where FTO (log2FC=− 1.61, adj-P=3.12×10^−23^), ALKBH5 (log2FC=− 0.73, adj-P=1.97×10⁻^17^), and YTHDF1 (log2FC=− 0.55, adj-P=3.65×10⁻^20^) were all significantly downregulated (Supplementary Figures S2 and S3).

Additional batch-correction diagnostics confirmed that ComBat substantially reduced, but did not completely eliminate, platform-associated variation. Beyond the PC1-associated variance reduction from 70.96% to 15.71%, the batch silhouette width decreased from 0.730 to − 0.078, PERMANOVA R2 for batch decreased from 0.867 to 0.004, and the median gene-level batch-associated R2 decreased from 85.11% to 0.11% across common genes. The condition-associated PERMANOVA R2 remained detectable (0.057 before ComBat and 0.052 after ComBat). These results support improved cross-platform harmonization while indicating that residual technical variation may remain (Supplementary Table S5 and Figures S14–S17).

To further address residual clinical heterogeneity, we summarized available metadata across cohorts and used available covariates for sensitivity analyses where possible. Because key variables such as harmonized SOFA/APACHE scores, lactate, pathogen information and standardized treatment exposure were not uniformly available, the results were interpreted with caution and residual confounding was explicitly retained as a limitation (Supplementary Table S2).

Univariate Cox regression analysis revealed that HNRNPC (HR=0.51, 95% CI 0.30–0.87, *P*=0.013) and ALKBH5 (HR=1.61, 95% CI 1.08–2.39, *P*=0.019) were significantly associated with 28-day mortality (Fig. 1E). Kaplan–Meier survival analysis indicated that patients in the high-expression group of YTHDF2 had significantly better survival rates than those in the low-expression group (Log-rank *P*=0.004); HNRNPC (*P*=0.011) and RBM15 (*P*=0.016) also showed similar trends (Supplementary Figure S4). Spearman's correlation analysis revealed that HNRNPC expression was negatively correlated with 28-day mortality (rho=− 0.12, adj-*P*=0.124), whereas WTAP and IGF2BP2 showed a positive trend in association with mortality (Fig. 1F). In a multivariate Cox regression analysis incorporating HNRNPC, ALKBH5, IGF2BP2, VIRMA, and YTHDF2, HNRNPC (HR=0.59, *P*=0.062) and ALKBH5 (HR=1.47, *P*=0.099) did not reach statistical significance, although they exhibited a visible trend toward prognostic association.

### Association between m6A regulators and glycolytic metabolism

**Fig. 2:**
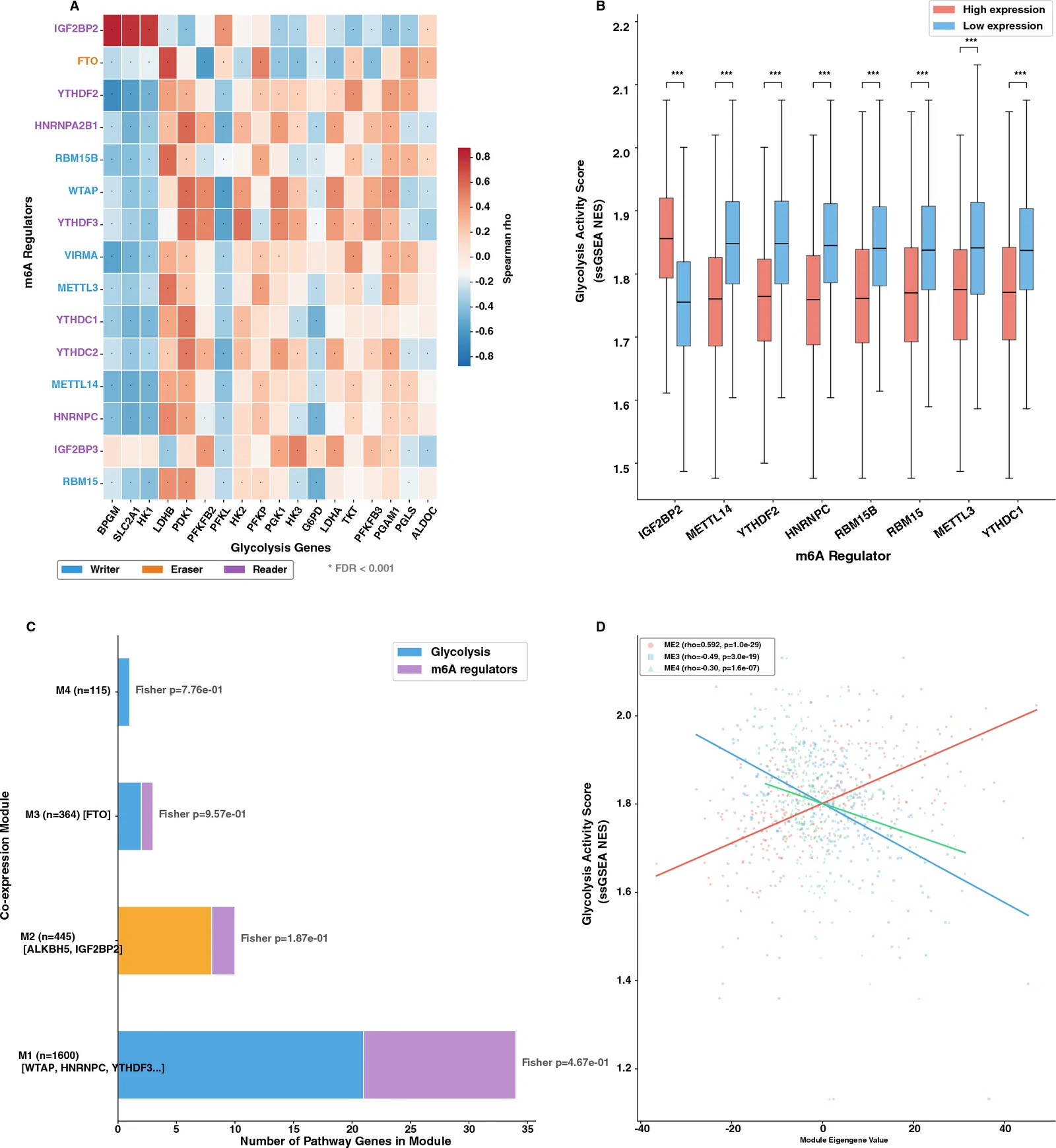
Associations between m6A regulators and glycolytic metabolism. **A** Spearman correlation heatmap between m6A regulators (rows) and glycolytic genes (columns); warm colors indicate positive and cool colors negative correlations, screened at FDR<0.001. **B** GSVA glycolytic-activity scores stratified by high versus low expression of each regulator; higher scores in the IGF2BP2- and ALKBH5-high groups indicate a positive association with glycolysis. **C** Composition of the four WGCNA co-expression modules, showing that ALKBH5 and IGF2BP2 co-localize in module M2. **D** Scatter plots of module eigengenes versus glycolytic activity; the steep positive slope for M2 (rho=0.592) and negative slope for the FTO-containing M3 identify the modules that drive the m6A–glycolysis axis. Readers should interpret module membership as shared co-regulation rather than direct physical interaction

In the correlation and pathway activity analyses in this section, we utilized all detectable m6A regulators from the GSE65682 training cohort, including METTL14, which was excluded from the cross-platform differential expression analysis due to limited probe coverage in the GSE95233 and GSE54514 platforms. Spearman's correlation analysis revealed extensive associations between m6A regulators and glycolytic genes (Fig. 2A). Under the strict screening criterion of FDR<0.001, multiple m6A–glycolysis gene pairs exhibited highly significant correlations. Among these, the positive correlation between IGF2BP2 and BPGM was the most prominent (rho=0.835, adj-P=4.3×10⁻^207^), followed by IGF2BP2-SLC2A1 (rho=0.784, adj-P=3.6×10⁻^165^) and IGF2BP2-HK1 (rho=0.759, adj-P=3.7×10⁻^149^). A strong positive correlation was also observed between FTO and LDHB (rho=0.687, adj-P=7.53×10⁻^111^), while YTHDF2 showed a strong negative correlation with BPGM (rho=− 0.658, adj-P=1.05×10⁻^98^) and SLC2A1 (rho=− 0.565, adj-P=2.76×10⁻^67^) (Supplementary Figure S5).

GSVA analysis revealed significant differences in glycolytic activity scores when grouped by high or low expression levels of m6A regulatory factors (Fig. 2B). The glycolytic activity in the high-expression group of IGF2BP2 was significantly higher than that in the low-expression group (effect size=0.109, adj-P=9.96×10⁻^39^), whereas the glycolytic activity in the high-expression groups of METTL14, YTHDF2, and HNRNPC was significantly lower than that in the low-expression groups. Notably, glycolytic activity was also higher in the ALKBH5 high-expression group than in the low-expression group (effect size=0.028, adj-P=1.84×10⁻^4^).

These results suggest that ALKBH5 and IGF2BP2 are positively associated with the m6A–glycolysis co-expression program; however, these module-level findings should be interpreted as associative evidence rather than direct proof of regulation.

WGCNA co-expression network analysis identified four co-expression modules (Fig. 2C). Module 2 (M2, containing 445 genes) was enriched for both ALKBH5 and IGF2BP2, two m6A regulatory factors, and included eight glycolysis-related genes. Analysis of module signature genes revealed that Module M2 showed the strongest positive correlation with glycolytic activity scores (rho=0.592, P=1.0×10⁻^29^, Fig. 2D), whereas Module M3 (containing FTO) exhibited a significant negative correlation with glycolytic activity (rho=− 0.49, P=3.0×10⁻^19^). Module 1 (M1, containing 13 m6A regulatory factors and 21 glycolysis genes) showed a weak negative correlation with glycolytic activity (rho=− 0.16, P=0.006). These results suggest that ALKBH5 and IGF2BP2 may play key positive regulatory roles in the m6A–glycolysis regulatory axis (Supplementary Figure S6).

### Overlap between ALKBH5-deficient transcriptomic effects and sepsis phenotypes

**Fig. 3:**
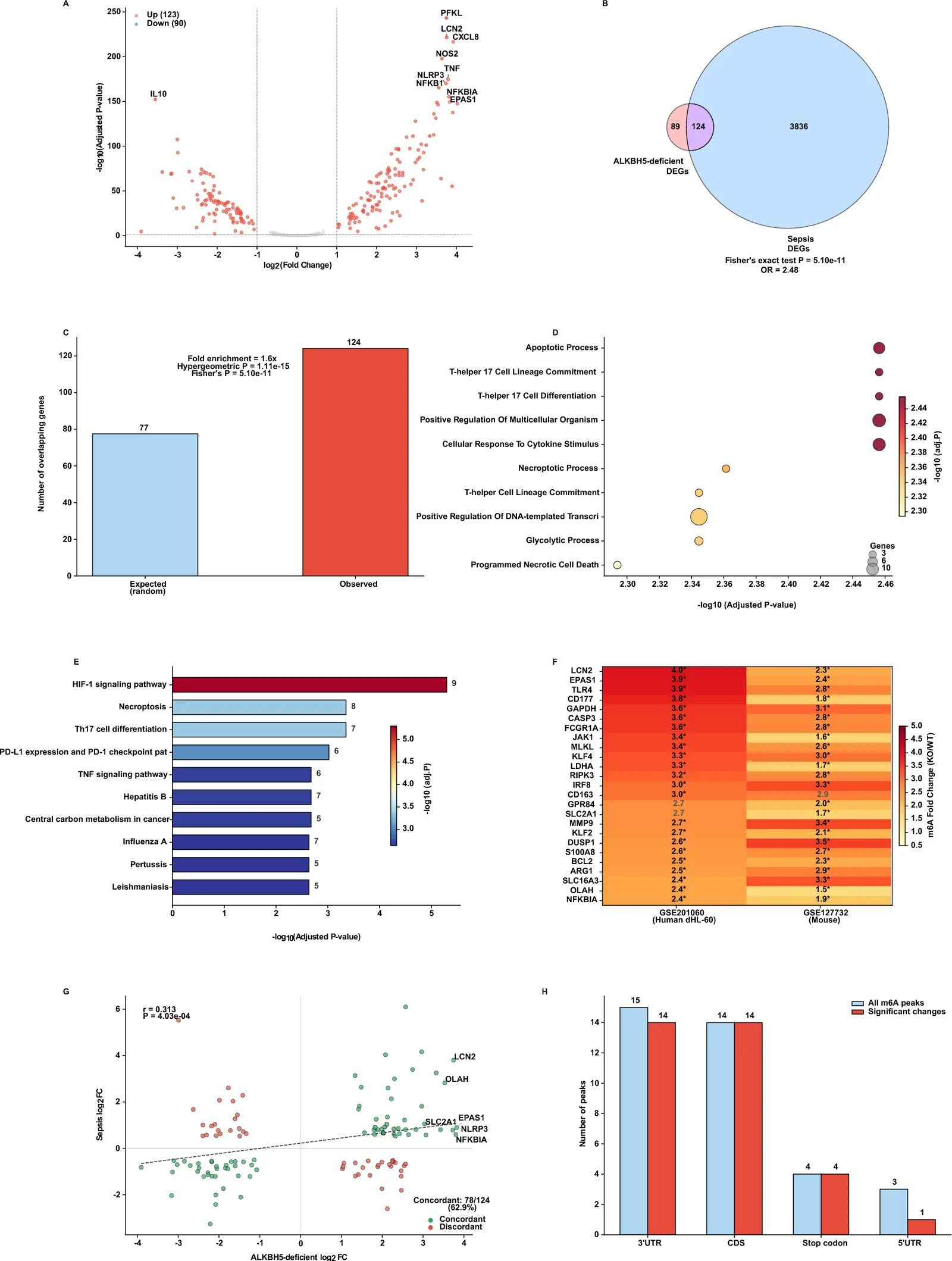
Overlap between the transcriptomic effects of ALKBH5 deficiency and sepsis features. **A** Volcano plot of differentially expressed genes after ALKBH5 knockout in macrophages (GSE198316). **B** Venn diagram and **C** overlap statistics comparing ALKBH5-knockout DEGs with sepsis DEGs; the observed overlap (124 genes) exceeds the random expectation (77 genes). **D** GO Biological Process and **E** KEGG pathway enrichment bubble plots of the overlapping genes (bubble size=gene count, color=adjusted P), highlighting glycolysis and HIF-1 signaling. **F** Heatmap of m6A2Target-validated ALKBH5 targets among the overlapping genes. **G** Scatter plot of directional concordance of expression changes between ALKBH5 deficiency and sepsis. **H** Genomic distribution of m6A peaks (3'UTR/CDS enrichment, DRACH motif). The panels collectively indicate that loss of ALKBH5 reproduces a glycolysis- and HIF-1-centered fraction of the sepsis transcriptome

To validate the role of ALKBH5 in the regulation of glycolysis during sepsis, we analyzed the transcriptome of ALKBH5-knockout macrophages (GSE198316). Differential expression analysis identified 213 significantly differentially expressed genes (123 upregulated, 90 downregulated, |log2FC|> 1, adj-P<0.05); a volcano plot revealed that immune and metabolic-related genes such as PFKL, LCN2, and CXCL8 were significantly upregulated (Fig. 3A). Cross-analysis of differentially expressed genes in ALKBH5-deficient cells with those in sepsis (3960 genes) revealed 124 overlapping genes, significantly higher than the random expectation of 77 (OR=2.48; Fisher’s exact test, *P*=5.10×10⁻^11^; hypergeometric test, *P*=1.11×10⁻^15^; Fig. 3B, C).

GO functional enrichment analysis of the 124 overlapping genes revealed that these genes were significantly enriched in the apoptosis process (GO:0006915, 9 genes, adj-P=0.003), Th17 cell differentiation (GO:0072539, 3 genes, adj-P=0.003), cytokine-stimulated response (GO:0071345, 11 genes, adj-P=0.003), and glycolysis (GO:0006096, 4 genes, including PFKFB2, LDHA, GAPDH, and HK2, adj-P=0.005, Fig. 3D). KEGG pathway analysis revealed that the HIF-1 signaling pathway was the most significant (9 genes, adj-P=5.06×10⁻^6^), followed by necrotic apoptosis (8 genes) and Th17 cell differentiation (7 genes; Fig. 3E, Supplementary Figure S7).

Directional consistency analysis revealed that among the 124 overlapping genes, 79 (63.7%) exhibited consistent expression change directions in ALKBH5 deficiency and sepsis (Fig. 3G). At the m6A modification level, analysis of the m6A2Target database revealed that several of the 124 overlapping genes have been previously identified as m6A targets of ALKBH5, including BCL2, CASP3, and KLF4 (Fig. 3F). GSE225143 MeRIP-seq data showed that following conditional knockout of METTL3, m6A modification levels were significantly reduced in multiple glycolytic genes (ENO1, GAPDH, HK2, LDHA, etc.). m6A peaks were primarily distributed in the 3′UTR and CDS regions and contained typical DRACH motifs (Fig. 3H, Supplementary Figure S8).

### Validation of ALKBH5 regulation of glycolytic genes in an independent experimental batch

**Fig. 4:**
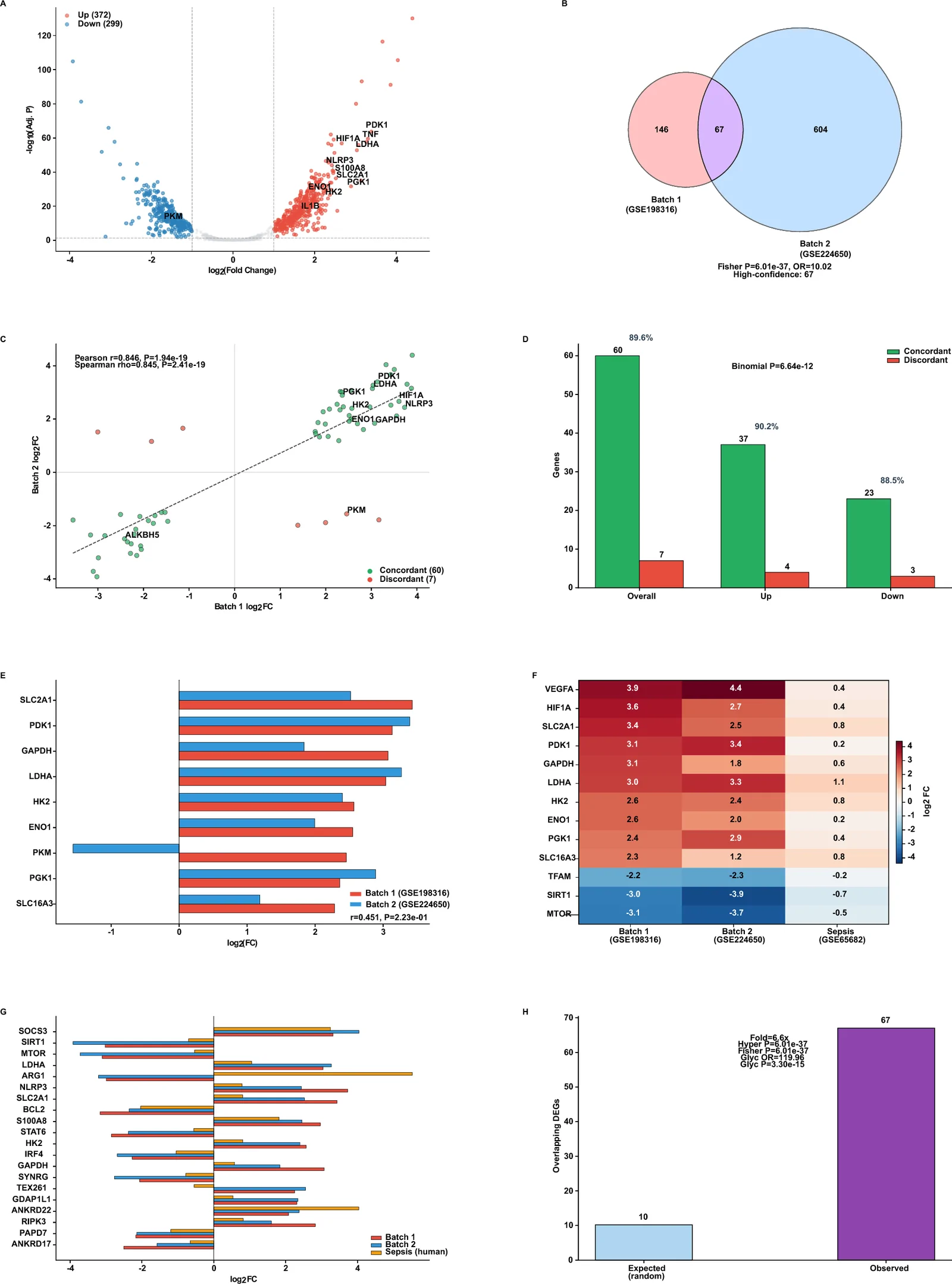
Independent-batch validation of ALKBH5 regulation of glycolytic genes. **A** Volcano plot of the independent ALKBH5-knockout batch (GSE224650). **B** Venn diagram of shared DEGs between the two batches and **C** scatter plot of inter-batch concordance, with **D** directional concordance statistics (89.6% agreement). **E** log2 fold-change comparison for key glycolytic genes across batches and **F** multi-batch heatmap. **G** Three-way comparison bar chart showing parallel up-regulation in both knockout batches and in sepsis. **H** Overlap statistics for high-confidence genes. The reproducibility across independent laboratories indicates that the ALKBH5–glycolysis association is robust rather than batch-specific

To enhance the reliability of our conclusions, we incorporated an independent experimental batch (GSE224650) for validation. Differential expression analysis identified 671 differentially expressed genes (372 upregulated, 299 downregulated; Fig. 4A). The two batches shared 67 high-confidence genes (Fisher’s OR = 10.02, P=6.01×10⁻^37^, Fig. 4B), with a consistency rate of 89.6% in the direction of expression changes between batches (60/67 genes showed consistent directional changes; binomial test P=6.64×10⁻^12^; Fig. 4C and D). Consistency reached 90.2% among upregulated genes (37/41) and 88.5% among downregulated genes (23/26).

Key glycolytic genes exhibited a highly consistent upregulation trend across both batches. SLC2A1 was upregulated 3.43-fold and 2.52-fold (log2FC) in batch 1 and batch 2, respectively; GAPDH was upregulated 3.07-fold and 1.84-fold; LDHA was upregulated 3.04-fold and 3.27-fold, respectively; HK2 was upregulated 2.57-fold and 2.40-fold, respectively; and PDK1 was upregulated 3.13-fold and 3.39-fold, respectively (Fig. 4E, F). Notably, these genes also exhibited an upregulated trend in sepsis patients, suggesting that the ALKBH5 knockout model can partially mimic the metabolic reprogramming observed in sepsis (Fig. 4G). A three-way cross-analysis (two batches of ALKBH5-KO versus sepsis) identified 29 core shared genes (Fig. 4H), including key glycolytic genes such as HK2, LDHA, GAPDH, SLC2A1, and SLC16A3 (Supplementary Figures S9 and S10).

### Validation of direct regulation of glycolytic genes by m6A modification

**Fig. 5:**
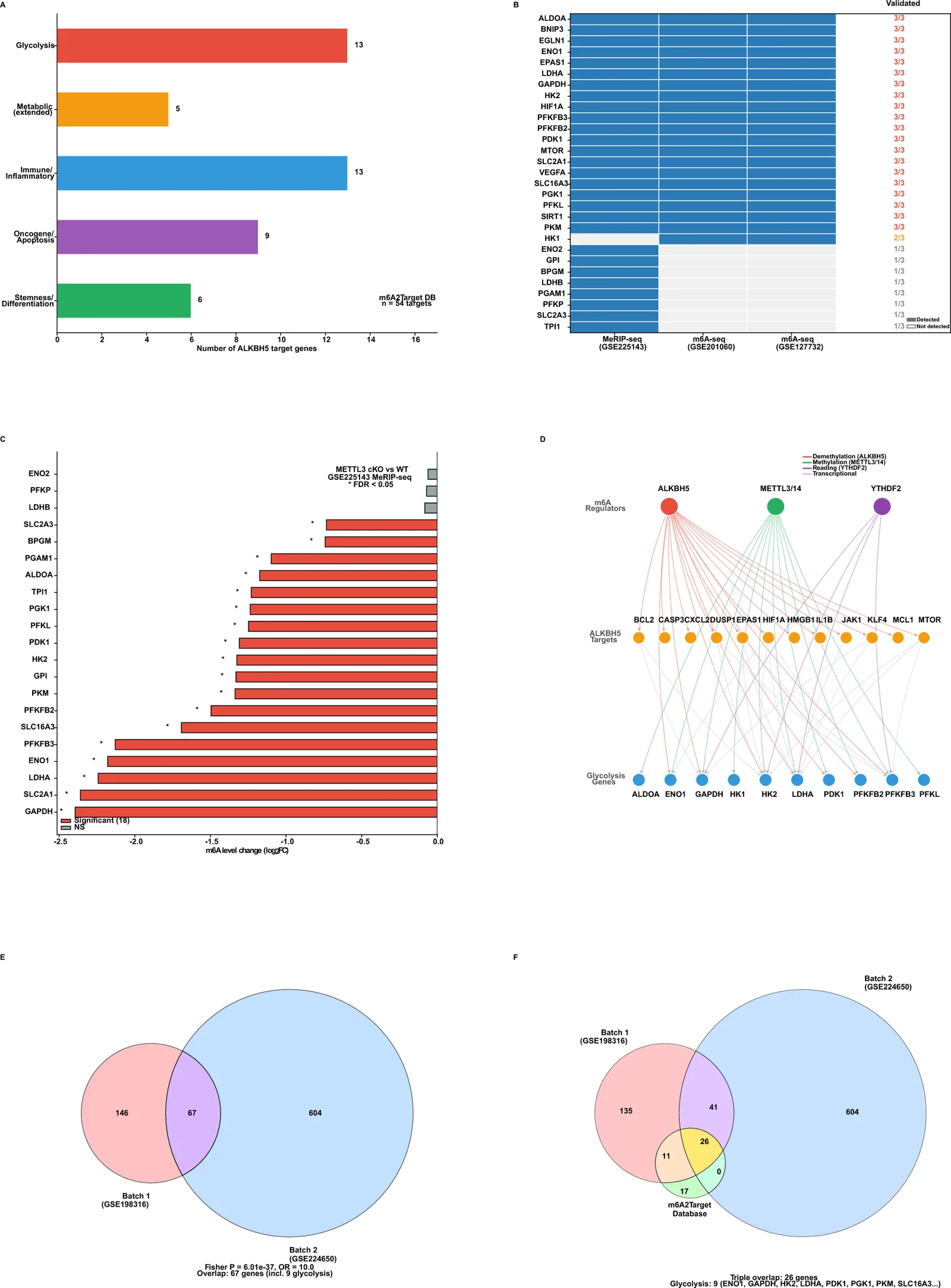
Multi-omics validation of m6A modification of glycolytic genes. **A** Functional classification of the 54 m6A2Target-validated ALKBH5 target genes. **B** Cross-dataset detection of m6A peaks across three MeRIP/m6A-seq datasets; genes detected in all three are high-confidence targets. **C** m6A-level changes after METTL3 knockout (GSE225143), with the largest reductions in GAPDH, SLC2A1, and LDHA. **D** Proposed ALKBH5–m6A–glycolysis regulatory network integrating writers, the eraser ALKBH5, and the reader YTHDF2. **E** Venn diagram and **F** three-way cross-validation identifying 26 high-confidence m6A targets, of which nine are core glycolytic genes. B-C should be read as epitranscriptomic evidence of m6A marking on glycolytic transcripts, complementary to the expression-level findings

To validate the direct regulation of glycolytic genes by m6A modifications at the epigenomic level, we integrated three independent m6A omics datasets. Analysis of the m6A2Target database revealed that among the 54 validated ALKBH5 target genes, multiple functional categories were covered, including glycolysis (13), immunity/inflammation (13), metabolic expansion (5), tumor/apoptosis (9), and stemness/differentiation (6) (Fig. 5A).

Cross-dataset MeRIP-seq validation revealed that m6A modification peaks were detected in a large number of glycolytic genes in at least two datasets (Fig. 5B). Among these, genes such as ALDOA, BNIP3, EGLN1, ENO1, GAPDH, HIF1A, HK2, LDHA, MTOR, and SLC2A1 were validated across all three datasets (3/3 datasets), indicating high-confidence m6A modifications. GSE225143 MeRIP-seq data revealed that following conditional knockout of METTL3, m6A modification levels were significantly reduced in 18 glycolytic genes (FDR<0.01), with the most pronounced changes observed in GAPDH (log2FC=− 2.39), SLC2A1 (log2FC=− 2.36), and LDHA (log2FC=− 2.24) (Fig. 5C). These m6A peaks were concentrated in the 3′UTR (15 peaks) and CDS (14 peaks) regions, all of which contained the typical DRACH motif (Supplementary Figure S11).

Based on the above multi-level evidence, we constructed an ALKBH5–m6A–glycolysis regulatory network (Fig. 5D). This network reveals that ALKBH5 regulates key target genes such as BCL2, CASP3, ENO1, GAPDH, HIF1A, HK2, and LDHA through demethylation, while METTL3/14 installs m6A marks on the same target genes via methylation, and YTHDF2 acts as an m6A reader to participate in the regulation of downstream mRNA degradation. Triple cross-validation (using two ALKBH5-KO batches and the m6A2Target database) identified 26 high-confidence m6A target genes (Fig. 5E, F), of which 9 (ENO1, GAPDH, HK2, LDHA, PDK1, PGK1, PKM, SLC16A3, and SLC2A1) are core genes of the glycolytic pathway, accounting for 34.6% (Fisher's exact test OR=119.96, *P*=1.30×10⁻^15^).

### Interactions between the immune microenvironment and metabolic pathways

**Fig. 6:**
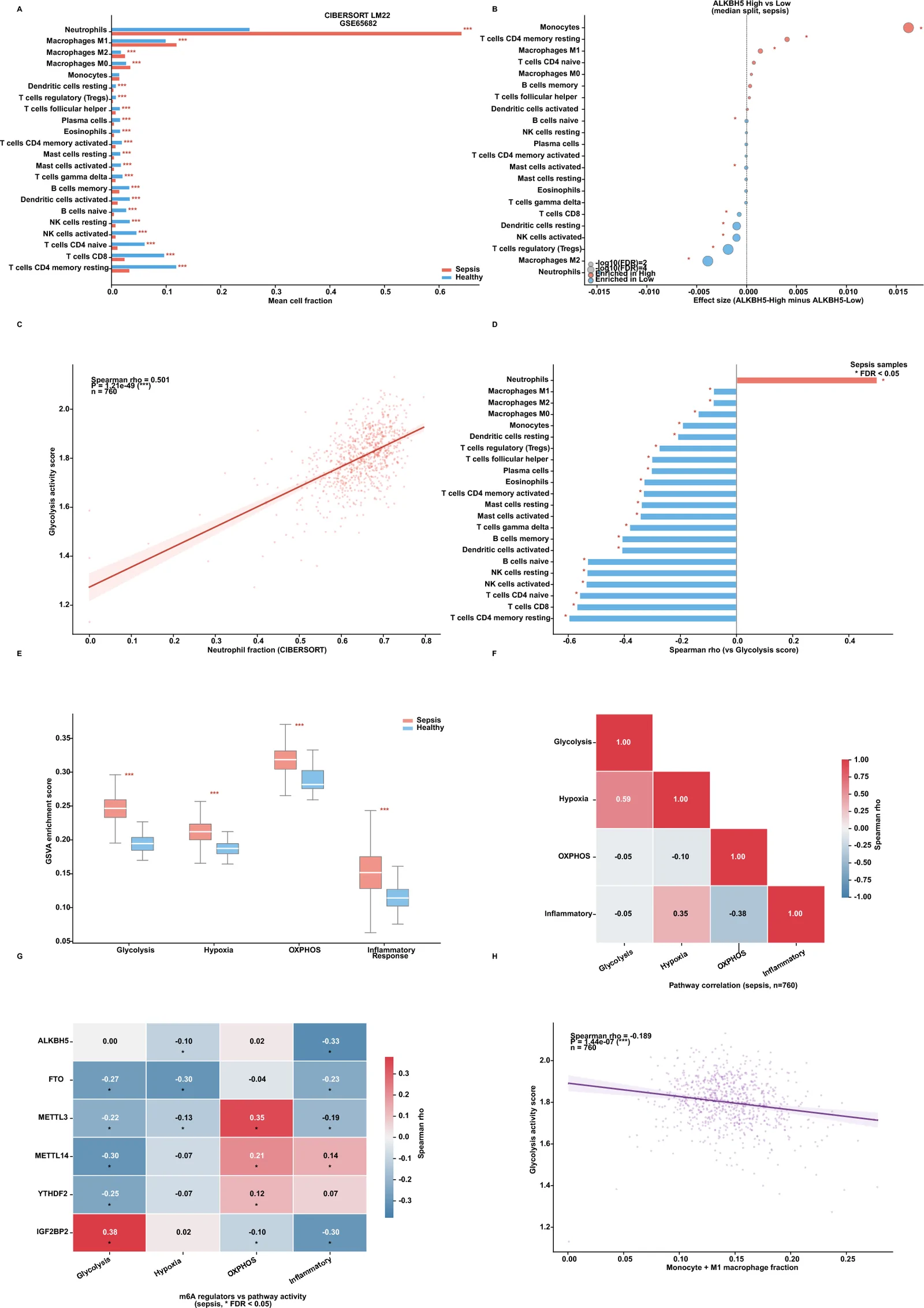
Interactions between the immune microenvironment and metabolic pathways. **A** CIBERSORT immune-cell composition in sepsis versus controls. **B** Immune differences between ALKBH5-high and ALKBH5-low groups. **C** Scatter plot of neutrophil abundance versus glycolytic activity (rho=0.501). **D** Correlations between each immune cell subset and glycolytic activity. **E** GSVA comparison of glycolysis, hypoxia, and oxidative-phosphorylation pathways. **F** Pathway–pathway correlation matrix. **G** Correlation between m6A regulators and metabolic pathways. **H** Monocyte/macrophage proportions versus glycolytic activity. The figure links innate-immune expansion to glycolytic reprogramming and situates ALKBH5/IGF2BP2 within this immunometabolic circuit

CIBERSORT immune deconvolution analysis revealed significant remodeling of the immune microenvironment in patients with sepsis (Fig. 6A). Compared with healthy controls, the proportion of neutrophils was significantly elevated in patients with sepsis (mean neutrophil proportion 0.64 vs. 0.25), while the proportions of CD4+ memory T cells and CD8+ T cells were significantly reduced. Differences in immune cell composition were also observed between the high- and low-ALKBH5 expression groups, with the high-ALKBH5 expression group exhibiting relatively higher proportions of monocytes and CD4+ memory T cells (Fig. 6B).

There was a strong positive correlation between the neutrophil proportion and the glycolytic activity score (Spearman's rho = 0.501, Fig. 6C), suggesting that changes in immune cell composition are closely associated with metabolic reprogramming. In sepsis samples, the proportions of neutrophils, monocytes, and M0 macrophages were positively correlated with glycolytic activity, whereas the proportions of CD4+ memory resting T cells, CD8+ T cells, and NK cells were negatively correlated with glycolytic activity (Fig. 6D). A significant negative correlation was also observed between the proportion of monocytes and M1 macrophages and glycolytic activity (rho=− 0.189, bottom right of Fig. 6H), further supporting the functional association between innate immune cell activation and glycolytic reprogramming.

GSVA metabolic pathway analysis revealed that glycolysis and hypoxia pathway scores were significantly higher in sepsis patients than in healthy controls, while oxidative phosphorylation pathway scores showed no significant difference (Fig. 6E). Correlation analysis among pathways indicated that the glycolysis pathway was highly positively correlated with the hypoxia pathway (rho=0.590), whereas associations with the oxidative phosphorylation and inflammatory response pathways were weaker (Fig. 6F). Correlation analysis between m6A regulators and metabolic pathways revealed that IGF2BP2 was positively correlated with the glycolysis pathway (rho=0.380) and negatively correlated with the inflammatory response pathway (rho=− 0.300); METTL3, METTL14, and YTHDF2 were negatively correlated with the glycolysis pathway and positively correlated with oxidative phosphorylation (bottom left of Fig. 6G, Supplementary Figure S12).

### Single-cell RNA-seq validation of the m6A–glycolysis association

**Fig. 7:**
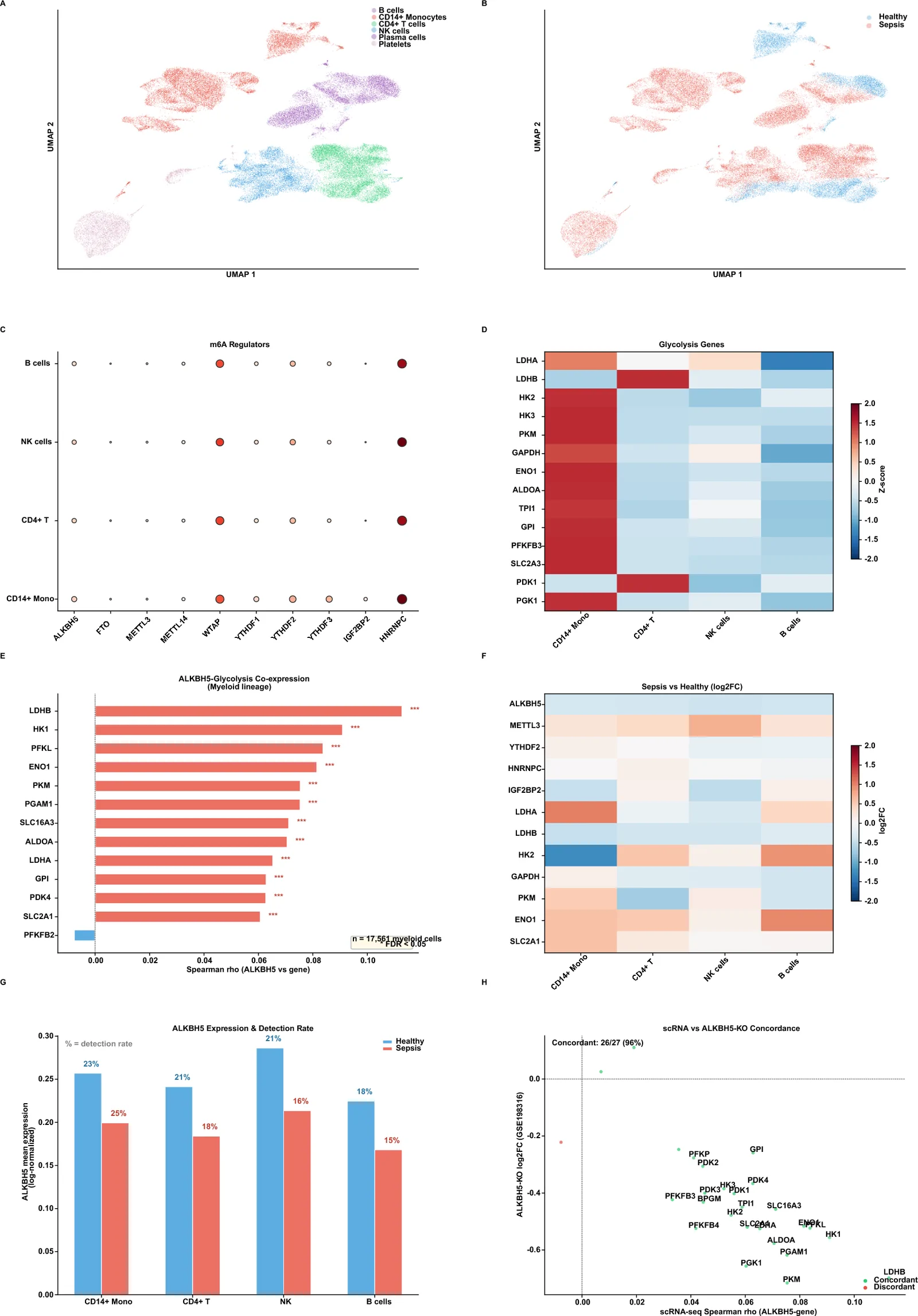
Single-cell RNA-sequencing analysis of the ALKBH5–glycolysis association. **A** UMAP embedding with annotated cell types from GSE147363 (38,559 cells). **B** UMAP colored by disease status (sepsis versus healthy). **C** Bubble plot of m6A-regulator expression across cell types (dot size=fraction expressing, color=mean expression). **D** Heatmap of glycolytic genes by cell type. **E** Patient-aggregated correlations between ALKBH5 and glycolytic genes in myeloid cells; correlation coefficients should be interpreted with attention to effect magnitude rather than statistical significance alone. **F** Heatmap of cell-type-specific differences. **G** Proportion of ALKBH5-positive cells by condition. **H** Concordance between single-cell correlations and bulk ALKBH5-knockout fold-changes (18/25 genes concordant). These panels provide supportive, cell-type-resolved evidence consistent with an ALKBH5-associated glycolytic program, while not establishing direct causality

The single-cell RNA sequencing dataset GSE147363 comprises 38,559 cells, which were annotated into eight major cell subpopulations via clustering analysis, including CD14+ monocytes, CD4+ T cells, NK cells, B cells, and neutrophils (Fig. 7A). UMAP visualization reveals significant differences in cell distribution between sepsis and healthy samples (Fig. 7B). Bubble plot analysis revealed that the expression of m6A regulators exhibits cell-type specificity across different cell subpopulations (Fig. 7C), with ALKBH5 showing relatively higher expression in CD14+ monocytes and neutrophils.

Glycolytic genes also exhibited differential expression patterns across different cell subsets. Heatmap analysis revealed that LDHA and HK2 were highly expressed in CD14+ monocytes, while LDHB was more abundant in B cells and NK cells (Fig. 7D). Further heatmap analysis revealed cell-type-specific expression differences between sepsis and healthy samples (Fig. 7F).In myeloid cells (CD14+ monocytes and neutrophils), ALKBH5 showed modest but statistically significant positive correlations with selected glycolytic genes, including LDHB (rho=0.113, adj-P=2.26×10^−49^), HK1 (rho=0.091, adj-P=1.92×10^−32^), and PFKL (rho=0.084, adj-P=8.95×10^−28^). These coefficients indicate weak effect sizes and should therefore be interpreted with attention to effect magnitude rather than statistical significance alone (Fig. 7E).

Under sepsis conditions, the proportion of ALKBH5-positive (expression>0) cells increased from 23.3% to 25.2% in CD14+ monocytes and decreased from 20.8% to 17.5% in CD4+ T cells, with statistically significant differences (Fig. 7G). Comparison of single-cell ALKBH5–glycolytic gene correlations with log2FC values from the ALKBH5-knockout model (GSE198316) showed that 26 of 27 glycolytic genes (96%) exhibited directionally concordant patterns (Fig. 7H). Here, concordance was defined as a positive ALKBH5–gene correlation in single-cell data accompanied by reduced expression after ALKBH5 knockout, or the reverse pattern. These results provide supportive, cell-type-resolved evidence for an ALKBH5-associated glycolytic program.To avoid overinterpreting weak single-cell correlations, we further summarized the concordance of ALKBH5-associated glycolytic signals across bulk transcriptomic association, WGCNA module-level evidence, ALKBH5-knockout perturbation, MeRIP/m6A-seq evidence, and patient-level single-cell correlations (Supplementary Table S3). Thus, the single-cell results provide supportive cell-type-resolved evidence for an ALKBH5-associated glycolytic program rather than definitive evidence of direct regulation (Supplementary Figure S13).

### Construction and validation of a prognostic model based on m6A regulatory factors

**Fig. 8:**
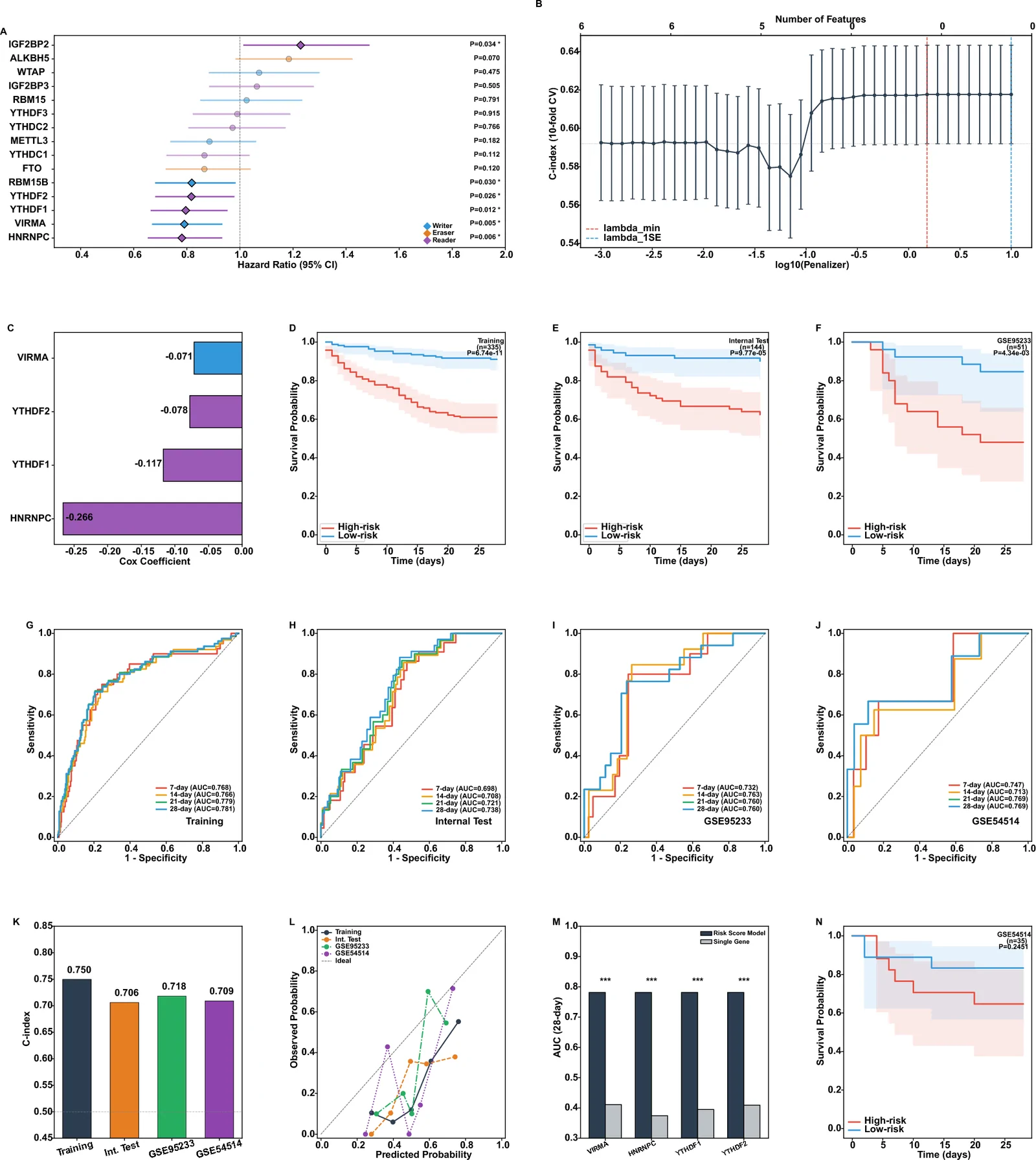
m6A-based prognostic model for 28-day mortality. **A** Univariate Cox forest plot of candidate regulators. **B** LASSO coefficient paths **C** cross-validated coefficient estimates retaining four genes (HNRNPC, YTHDF1, YTHDF2, VIRMA). **D**-**F** Kaplan–Meier curves separating high- and low-risk groups in the training set, internal validation set, and GSE95233. **G**-**J** Time-dependent ROC curves at 7/14/21/28 days. **K** C-index comparison across cohorts. **L** Calibration curves comparing predicted and observed survival. **M** Model versus single-gene 28-day AUC. **N** Validation in GSE54514, where separation did not reach significance (log-rank *P*=0.245). This discrepancy indicates incomplete external reproducibility and highlights the need for prospective validation in larger cohorts with harmonized sampling time, platform processing and clinical annotations

Univariate Cox regression was first performed to identify m6A regulators associated with 28-day mortality（Fig. 8A）. Candidate prognostic regulators were subsequently entered into LASSO-Cox regression with ten-fold cross-validation to reduce model complexity and construct the final prognostic signature (Fig. 8B, C). The final model retained four m6A regulators, including HNRNPC, YTHDF1, YTHDF2, and VIRMA. The corresponding coefficients are shown in Figure 8C.

The risk score formula is: Risk Score = −0.266 × HNRNPC − 0.117 × YTHDF1 − 0.078 × YTHDF2 − 0.071 × VIRMA. In the training set, the 28-day survival rate in the high-risk group was significantly lower than that in the low-risk group (Log-rank P=6.74×10^−11^, Fig. 8D), and this difference was validated in the internal validation set (*P*=9.77×10^−5^, Fig. 8E). Significant stratification was also observed in the external validation set GSE95233 (*P*=4.34×10^−3^, Fig. 8F). However, the external validation cohort GSE54514 did not reach statistical significance by log-rank testing (*P*=0.245, Fig. 8N), indicating incomplete external reproducibility.

Time-dependent ROC analyses were used to evaluate the predictive performance of the model at 7, 14, 21, and 28 days across the training and validation cohorts (Fig. 8G–J). The C-index was 0.750 in the training set, 0.706 in the internal validation set, 0.718 in GSE95233, and 0.709 in GSE54514 (Fig. 8K). Calibration analysis was used to assess the agreement between predicted and observed survival probabilities (Fig. 8L), and comparison with single-gene models suggested that the combined four-gene signature provided better overall prognostic information than individual m6A regulators alone (Fig. 8M).Thus, GSE54514 did not represent a complete absence of predictive signal, but rather a cohort in which dichotomized Kaplan–Meier stratification was insufficiently robust to reach statistical significance. We therefore interpret the four-gene model as a preliminary prognostic framework that requires prospective validation (Supplementary Table S4).

## Discussion

This study integrates multiple sepsis transcriptome cohorts, ALKBH5 functional datasets, m6A modification datasets, and single-cell RNA sequencing data. To our knowledge, it is the first systematic analysis of the expression patterns, potential functional roles, and possible regulatory mechanisms of m6A RNA modification regulators in the context of glycolytic metabolic reprogramming associated with sepsis. We found that most m6A regulators were significantly downregulated in the peripheral blood of septic patients, particularly the methyltransferase METTL3 and the reader protein HNRNPC. Conversely, the m6A readers IGF2BP2 and IGF2BP3 were significantly upregulated. This altered expression pattern may reflect a global remodeling of the m6A epitranscriptome in sepsis. Downregulation of methyltransferases such as METTL3 may lead to a global reduction in m6A modification, thereby affecting the stability and translational regulation of a large number of mRNAs. The upregulation of IGF2BP2 warrants particular attention, as IGF2BP2 has been shown to stabilize its target mRNAs in an m6A-dependent manner [33]. Its elevated expression in sepsis may represent a compensatory response to reduced m6A modifications, aiming to preserve the stability of key immune and metabolic gene transcripts. These findings align with recent studies on epigenetic regulation in sepsis, suggesting that disrupted epitranscriptomic regulation may represent a crucial upstream mechanism underlying immune and metabolic dysregulation in sepsis [34].

In response to the reviewer concern regarding causal interpretation, we have revised the manuscript to emphasize that the ALKBH5–m6A–glycolysis relationship should be regarded as a biologically plausible, hypothesis-generating model derived from integrated public data rather than a validated mechanistic pathway. The current evidence supports association and directional concordance across multiple layers, but does not establish that ALKBH5 is an upstream causal regulator of glycolytic reprogramming in sepsis.

One of the core findings of this study is the potential involvement of ALKBH5, an m6A demethylase, in glycolytic reprogramming during sepsis. WGCNA analysis revealed that ALKBH5 and IGF2BP2 co-localize within a co-expression module that is most strongly positively correlated with glycolytic activity (rho=0.592), suggesting possible synergistic roles in the post-transcriptional regulation of glycolytic genes. Analysis of the ALKBH5 knockout model further supported this hypothesis: genes differentially expressed due to ALKBH5 deficiency showed highly significant overlap with sepsis-associated differentially expressed genes (OR=2.48, *P*=5.10×10⁻^11^), and these shared genes were enriched in the HIF-1 signaling pathway and glycolytic processes. Independent experimental batches validated 89.6% directional consistency (binomial test *P*=6.64×10⁻^12^), underscoring the robustness of the findings. At the molecular level, ALKBH5 may modulate mRNA stability and translation efficiency by demethylating m6A marks on key glycolytic enzyme genes. MeRIP-seq data suggested that core glycolytic genes such as ENO1, GAPDH, HK2, LDHA, and SLC2A1 are indeed associated with m6A modifications, with m6A peaks concentrated at DRACH motifs in the 3′UTR and CDS regions. Previous studies indicate that m6A modifications in the 3′UTR region are typically recognized by YTHDF2 and mediate mRNA degradation [20]. Therefore, removal of m6A marks by ALKBH5 at these sites may enhance the stability of glycolytic gene mRNAs, thereby promoting the expression of glycolytic enzymes and upregulating glycolytic activity. This mechanism is consistent with the metabolic reprogramming needs of immune cells in early sepsis, namely the rapid upregulation of glycolysis to meet the energy requirements of the inflammatory response [8, 35 ]. Because these inferences rest on integrated transcriptomic and epitranscriptomic correlations rather than direct perturbation in vivo, we interpret ALKBH5 as a candidate driver of, rather than a proven causal switch for, glycolytic reprogramming in sepsis.

Analysis of the immune microenvironment revealed a close relationship among m6A, glycolysis, and immunity. CIBERSORT analysis showed a significantly elevated proportion of neutrophils in sepsis patients, which was strongly positively correlated with glycolytic activity (rho = 0.501). These findings are consistent with previous reports of neutrophil metabolic reprogramming in sepsis [36]. Notably, the proportions of monocytes and M1 macrophages also showed a positive correlation with glycolytic activity, further supporting the notion that activation of innate immune cells relies on glycolytic metabolism [37]. Single-cell RNA sequencing analysis validated these findings at a higher resolution: ALKBH5 exhibited significant positive correlations with glycolytic genes such as TPI1, PFKL, and PGAM1 in CD14+ monocytes, and the proportion of ALKBH5-positive cells significantly increased under septic conditions. These results suggest that ALKBH5 may play a particularly important metabolic regulatory role within the monocyte/macrophage lineage. Given that monocytes/macrophages are core effector cells in the sepsis immune response [38], ALKBH5-associated m6A regulation may critically influence the metabolic fate (glycolysis versus oxidative phosphorylation) of these cells, thereby potentially affecting pro-inflammatory or immune-tolerant phenotypes. Metabolic pathway analysis further revealed a strong positive correlation between glycolysis and hypoxia pathways (rho = 0.590), with HIF-1 signaling emerging as the top enriched pathway among the overlapping genes from the ALKBH5 knockout analysis. As the core transcription factor linking hypoxic response and glycolytic metabolism [39], HIF-1α mRNA itself appeared to be associated with m6A modification (m6A peaks detected in all three MeRIP-seq datasets). This suggests that ALKBH5 might indirectly amplify overall glycolytic pathway activation by influencing HIF-1α mRNA stability through m6A modification.

We also acknowledge that several single-cell correlations between ALKBH5 and individual glycolytic genes were modest in magnitude despite highly significant adjusted P-values. These associations should not be interpreted as strong single-gene effects or as direct evidence of ALKBH5-mediated regulation. Their biological relevance lies primarily in their directional consistency with independent lines of evidence, including bulk transcriptomic associations, WGCNA module-level enrichment, ALKBH5-knockout transcriptomic changes, MeRIP/m6A-seq evidence of m6A marking on glycolytic transcripts, and concordance between single-cell correlations and knockout-derived fold-changes. Thus, the weak but consistent single-cell correlations support a cell-type-resolved ALKBH5-associated glycolytic program, while the causal relationship remains hypothesis-generating and requires direct experimental validation.

The four-gene m6A prognostic model (HNRNPC, YTHDF1, YTHDF2, VIRMA) showed promising but incomplete external reproducibility. Significant survival stratification was observed in the training set, internal validation set, and GSE95233, whereas the GSE54514 cohort did not reach statistical significance by log-rank testing (*P*=0.245). This inconsistency should not be attributed solely to the small sample size, although the sepsis patient number in GSE54514 was limited (*n*=35). Other factors may also have contributed, including cohort-specific disease biology, differences in microarray platforms, temporal sampling variation, heterogeneity in infection source and organ dysfunction, and potential instability of the four-gene signature across clinical contexts[40]. Therefore, the current model should be regarded as a preliminary prognostic framework rather than a clinically applicable prognostic tool. Future studies should validate this signature prospectively in larger, multicenter cohorts with harmonized sampling time points, complete clinical annotations, and direct comparison with established bedside severity scores such as SOFA, APACHE II, lactate, and procalcitonin.

This study has several limitations that warrant clarification. First, all analyses were based on retrospective data from public databases and require validation through prospective cohort studies. Second, functional validation of ALKBH5 relied on in vitro knockout models, and its potential role in glycolytic reprogramming during sepsis has not been directly demonstrated in animal models. Moreover, existing datasets predominantly originate from macrophage cell lines, so caution is warranted when extrapolating these findings to whole-blood samples from sepsis patients. Third, validation of m6A modifications primarily relies on MeRIP-seq data, which cannot precisely determine modification sites at single-nucleotide resolution. Future next-generation m6A detection technologies, such as miCLIP-seq or DART-seq, may provide more accurate information on modification sites [41]. Fourth, this study focuses on m6A regulator expression at the whole-blood transcriptome level and cannot distinguish the differential contributions of m6A modifications across distinct immune cell subpopulations, although single-cell analyses partially address this limitation. Fifth, the external validation sample size for the prognostic model is relatively limited, particularly in the sepsis patient cohort of GSE54514 (*n*=35). Large-scale, multicenter prospective validation studies will be essential to assess the model's clinical utility. Sixth, because sepsis is a dynamic and clinically heterogeneous syndrome, residual confounding by disease stage, infection source, severity, and treatment cannot be excluded, and the integrated cohorts spanned different platforms and sepsis definitions. Several caveats intrinsic to large-scale omics analyses temper the interpretation of our findings. The pronounced imbalance between sepsis cases and healthy controls in GSE65682 (760 versus 42) and the very large cell numbers in the single-cell data can render biologically modest effects statistically significant; accordingly, we applied Benjamini–Hochberg false-discovery-rate control, emphasized effect sizes alongside P-values, and refrained from inferring functional relationships from weak correlations (for example, |rho|< 0.3). Because ComBat-adjusted values no longer represent raw biological measurements and may partially intermix technical and biological variance, the present results should be regarded as exploratory observations that require confirmation in reproducible single-patient datasets and experimental cell-based systems.

Residual confounding remains an important limitation of this study. Although we summarized available clinical metadata and performed sensitivity analyses using reported variables where possible, the integrated cohorts differed in platform, sampling design, sepsis definition, and available clinical annotation. Key variables such as harmonized SOFA/APACHE scores, lactate, procalcitonin, infection source, causative pathogen, organ dysfunction pattern, and treatment exposure were not uniformly available. Therefore, unmeasured confounders may influence associations among ALKBH5 expression, glycolytic activity, immune composition, and clinical outcomes.

A further limitation is the integration of gene-expression data generated from multiple independent microarray platforms. Although ComBat correction substantially reduced platform-associated variation according to multiple metrics, including the PC1-associated variance reduction from 70.96% to 15.71%, kBET, silhouette analysis, PERMANOVA, and gene-level variance decomposition, batch correction cannot fully disentangle technical variation from true cohort-specific biological heterogeneity. Residual technical variability may persist, particularly at the level of individual genes whose probe-specific hybridization characteristics differ across platforms. Thus, the integrated analyses should be interpreted as exploratory and require validation in prospectively collected cohorts processed using standardized platforms or RNA sequencing.

Despite the aforementioned limitations, this study systematically integrates transcriptomic, epitranscriptomic, perturbation and single-cell omics data to explore the potential association of m6A modifications with glycolytic reprogramming during sepsis. The findings provide a hypothesis-generating framework for future epitranscriptomic and immunometabolic studies in sepsis. Future research directions include validating the effects of ALKBH5 perturbation in sepsis-relevant animal and cellular models, conducting direct m6A-site and glycolytic-function assays, and validating the preliminary prognostic framework in prospective, multicenter cohorts with harmonized clinical annotation.

## Supplementary Information


Supplementary Material 1.Supplementary Material 2.Supplementary Material 3.Supplementary Material 4.Supplementary Material 5.Supplementary Material 6.

## Data Availability

All datasets analyzed are publicly available from the Gene Expression Omnibus (GEO; https://www.ncbi.nlm.nih.gov/geo/) under accession numbers GSE65682, GSE95233, GSE54514, GSE198316, GSE224650, GSE225143, GSE201060, GSE127732, and GSE147363. The m6A2Target database (http://m6a2target.canceromics.org/) was used for validated target genes. Processed data and analysis code generated in this study are available from the corresponding author upon reasonable request.
