# Supplementary material for: Role and prognostic value of N6-methyladenosine RNA modification regulators in glycolytic reprogramming during sepsis
Source: J Intensive Care. 2026 Jul 30;14:90. doi: 10.1186/s40560-026-00920-4 (PMC13528130; doi:10.1186/s40560-026-00920-4)
Supplement: Supplementary file 1 [file 40560_2026_920_MOESM1_ESM.docx]

**Supplementary material**

**Figure S1.** Details of data quality control, including the PCA distributions and sample quality metrics before and after batch correction.

**Figure S2.** Survival analysis of m6A regulatory factors in the validation cohort (GSE95233).

**Figure S3.** Violin plots showing the expression distributions of all 15 m6A regulatory factors in sepsis patients and healthy controls.

**Figure S4.** Kaplan–Meier survival curves for each m6A regulatory factor.

**Figure S5.** Detailed correlation analysis between m6A regulatory factors and glycolysis genes.

**Figure S6.** Detailed WGCNA settings

**Figure S7.** Extended pathway enrichment analysis of genes overlapping with ALKBH5 loss.

**Figure S8.** Detailed analysis of differentially expressed genes in the ALKBH5 knockout model.

**Figure S9.** DEGsin the second batch cohort (GSE224650).

**Figure S10.** Cross-batch validation details of metabolism-related genes.

**Figure S11.** Expanded analysis of m6A modification

**Figure S12.** Extended results of the immune microenvironment analysis

**Figure S13.** Extended analysis results of single-cell RNA sequencing.

## **Figure S14:** PCA before/after ComBat colored by dataset and condition.

PCA plots before and after ComBat correction. Samples are coloured by dataset/platform and biological condition. Before correction, samples clustered primarily by dataset; after correction, dataset-driven separation was markedly reduced. Condition-associated structure remained partially preserved.

## **Figure S15:** Gene-level variance decomposition before and after ComBat.

## Gene-level variance decomposition across common genes before and after ComBat correction. The median variance attributable to batch decreased substantially, whereas condition-associated variance remained detectable but attenuated.

**Figure S16:** Biological signal preservation; emphasizes strong GSE65682/GSE95233 and weaker GSE54514.

Biological signal preservation after ComBat correction. Within-dataset logFC correlations were high in GSE65682 and GSE95233, whereas GSE54514 showed weaker stability. Cross-dataset concordance remained strong between GSE95233 and GSE65682 but was poor for comparisons involving GSE54514, supporting a cautious interpretation of residual cohort heterogeneity.

**Figure S17:** PERMANOVA and silhouette metrics.

Quantitative assessment of batch-effect reduction and biological signal preservation. PERMANOVA R² and silhouette width for batch labels decreased markedly after ComBat, while condition-associated signal remained detectable.
